# Dual Role of G-runs and hnRNP F in the Regulation of a Mutation-Activated Pseudoexon in the Fibrinogen Gamma-Chain Transcript

**DOI:** 10.1371/journal.pone.0059333

**Published:** 2013-03-22

**Authors:** Valeria Rimoldi, Giulia Soldà, Rosanna Asselta, Silvia Spena, Cristiana Stuani, Emanuele Buratti, Stefano Duga

**Affiliations:** 1 Dipartimento di Biotecnologie Mediche e Medicina Traslazionale, Università degli Studi di Milano, Milan, Italy; 2 International Centre for Genetic Engineering and Biotechnology (ICGEB), Trieste, Italy; CNRS UMR7275, France

## Abstract

Most pathological pseudoexon inclusion events originate from single activating mutations, suggesting that many intronic sequences are on the verge of becoming exons. However, the precise mechanisms controlling pseudoexon definition are still largely unexplored. Here, we investigated the *cis*-acting elements and *trans*-acting regulatory factors contributing to the regulation of a previously described fibrinogen gamma-chain (*FGG*) pseudoexon, which is activated by a deep-intronic mutation (IVS6-320A>T). This pseudoexon contains several G-run elements, which may be bound by heterogeneous nuclear ribonucleoproteins (hnRNPs) F and H. To explore the effect of these proteins on *FGG* pseudoexon inclusion, both silencing and overexpression experiments were performed in eukaryotic cells. While hnRNP H did not significantly affect pseudoexon splicing, hnRNP F promoted pseudoexon inclusion, indicating that these two proteins have only partially redundant functions. To verify the binding of hnRNP F and the possible involvement of other *trans*-acting splicing modulators, pulldown experiments were performed on the region of the pseudoexon characterized by both a G-run and enrichment for exonic splicing enhancers. This 25-bp-long region strongly binds hnRNP F/H and weakly interacts with Serine/Arginine-rich protein 40, which however was demonstrated to be dispensable for *FGG* pseudoexon inclusion in overexpression experiments. Deletion analysis, besides confirming the splicing-promoting role of the G-run within this 25-bp region, demonstrated that two additional hnRNP F binding sites might instead function as silencer elements. Taken together, our results indicate a major role of hnRNP F in regulating *FGG* pseudoexon inclusion, and strengthen the notion that G-runs may function either as splicing enhancers or silencers of the same exon.

## Introduction

Splice-site selection in higher eukaryotes depends on multiple parameters such as splice-site strength, presence or absence of activating and inhibitory regulatory elements, RNA secondary structure, and gene architecture [1]. The relative contribution of each of these components controls how efficiently splice sites are recognized and flanking introns are removed. In particular, every exon has its specific set of identity elements that permit its recognition by the spliceosome, a “splicing code” that precisely defines the overall binding affinity for the splicing machinery [2], [3]. While the first layer of this code, namely the consensus splice sites, is relatively easy to identify, the additional layers are composed of highly degenerated signals that act in a complex combinatorial way and are much more difficult to decipher. Indeed, an array of diverse intronic and exonic splicing enhancers (ISEs and ESEs) and silencers (ESSs and ISSs) serve as binding sites for specific *trans*-acting regulatory factors to the pre-mRNA, and are thus required either to direct the splicing machinery to the appropriate sites or to inhibit the use of potential cryptic splice sites. ESEs, in particular, appear to be widespread, and might be present in most, if not all, exons, including constitutive ones. The best characterized ESEs promote splicing by interacting with members of the serine/arginine-rich (SR) protein family [4]. ESE motifs are quite degenerated and often overlapping, making them difficult to predict on the basis of the nucleotide sequence alone. For instance, analysis of SR-protein binding motifs showed that the major family members recognize fairly degenerated consensus sequences, varying from 5 to 7 nucleotides with a high purine content [5].

Silencing elements are less well characterized than ESEs, and their mechanisms of action are still not fully understood. The genetic context seems to be extremely important in determining the effect of both ESSs and ISSs. A well-established regulatory motif consisting of a stretch of three or more guanine nucleotides, the so called “G-run” element, may function both as an ESS and as an ISE, depending on its position. Indeed, it can cause exon skipping when placed within an exon, but it can also promote exon inclusion when located downstream of a weak 5′ splice site [6]. Both ESSs and ISSs work by interacting with negative regulators, which often belong to the heterogeneous nuclear ribonucleoprotein (hnRNP) family. In particular, the hnRNP I protein (also known as polypyrimidine-tract-binding protein, PTB) and proteins of the hnRNP A/B and hnRNP H families are among the best-characterized mediators of silencing [4].

Despite the efforts to classify general splicing regulatory sequences and their binding factors, exceptions are not uncommon: classical SR proteins are known to be involved in splicing repression in few cases [7], whereas some well-characterized hnRNP proteins may also act as splicing enhancers [8], [9]. Therefore, experimental studies are required to clarify the role played by even well-known splicing factors in each specific gene context.

Deciphering the splicing code is becoming increasingly important for the characterization of pathogenic mechanisms leading to human disease, as up to 60% of disease-causing mutations are found to affect splicing [10], [11]. In general, changes in splicing *cis*-regulatory elements can lead to exon skipping, intron retention, creation of ectopic splice sites, or activation of cryptic ones [12], [13], [14]. Another important pathological outcome of splicing mutations, which has been long overlooked, is the activation of pseudoexons. Despite the abundance of potential pseudoexons (50–200 nt-long intronic sequences with apparently viable splice sites at either end), their inclusion during normal pre-mRNA processing seems rare, although it has been described to occur as a regulatory mechanism for the expression of specific genes [15]. However, the actual frequency of pseudoexon activation might be underestimated due to nonsense-mediated-mRNA degradation of transcripts carrying out-of-frame pseudoexons. Most mutation-induced pseudoexon inclusion events originate from a single activating mutation, suggesting that many intronic sequences might be poised on the brink of becoming exons [16]. These mutations generally involve the creation of *de novo* functional donor or acceptor splice sites within an intronic sequence, followed by the subsequent selection of nearby “opportunistic” acceptor or donor sites. Alternatively, other frequent mechanisms leading to pseudoexon activation involve the creation of enhancer or loss of silencer splicing regulatory elements. Conversely, the *trans*-acting factors involved in pseudoexon inclusion are less known, although hnRNP proteins seem to have an important modifier role [16].

We previously described a deep-intronic homozygous mutation (IVS6-320A>T) that causes the inclusion of a 75-bp pseudoexon between exons 6 and 7 of the fibrinogen gamma-chain gene (*FGG*) transcript in a patient affected by congenital afibrinogenemia [17]. This mutation reinforces a pre-existing cryptic donor splice site by extending its complementarity to U1snRNA, eventually resulting in the activation of a pseudoexon. We also suggested that, apart from the cryptic splice-site activation, the modulation of normally silent regulatory elements could also play a role in this mutation-induced pseudoexon inclusion [17]. In the present work, we address this issue by functionally dissecting both the *cis*-acting elements and the *trans*-acting regulatory factors that contribute to the regulation of this pseudoexon insertion event.

## Results

**Figure 1:**
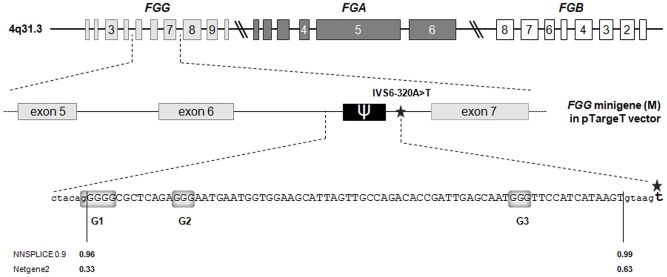
Schematic representation of the 75-bp *FGG* pseudoexon activated by the IVS6-320A>T mutation. (top) The fibrinogen cluster; boxes and lines represent exons and intronic/intergenic regions, respectively (only exons are drawn to scale); the two parallel slanted lines indicate breaks in the scale. (middle) The *FGG* minigene (M) cloned in pTargeT vector; the star marks the IVS6-320A>T mutation. (bottom) The complete 75-bp-long pseudoexon sequence and flaking splice sites; nucleotides belonging to the pseudoexon are in capital letters; the strength of pseudoexon splice sites, calculated by using the NNSPLICE 0.9 (http://www.fruitfly.org/seq_tools/splice.html) and the Netgene2 (http://www.cbs.dtu.dk/services/NetGene2/) software is reported below the corresponding sequence; G-stretches are shaded in gray.

A previous work from our group demonstrated that a single nucleotide substitution within intron 6 of the *FGG* gene (IVS6-320A>T) results in the inclusion of a disease-causing pseudoexon in nearly the totality of mature transcripts (Figure 1) [17]. This nucleotide substitution produces an extended complementarity to U1snRNA at a cryptic donor splice site. However, the exiguity of residual wild-type splicing, as well as the existence in other *FGG* exons (i.e. exons 3 and 9) of physiologic donor splice sites with sequence similar to the cryptic one -which is totally neglected by the splicing machinery in the wild-type context- suggested the existence of splicing regulatory mechanisms modulating the inclusion of this pseudoexon (Figure S1A). This prompted us to investigate in more detail the in-*cis* and in-*trans* elements involved in this pseudoexon activation/suppression.

### hnRNP F Regulates Pseudoexon Inclusion in the FGG mRNA

**Figure 2:**
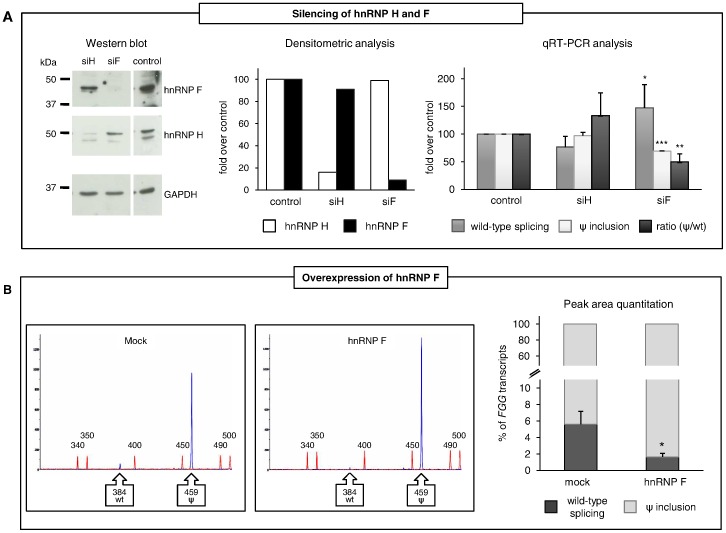
Effect of hnRNP H and F modulation on the regulation of *FGG* pseudoexon splicing. (A) Knockdown of hnRNP H and F. Western blot (left) and corresponding densitometric analysis (middle) demonstrating the actual silencing of hnRNP H and F proteins in RNAi experiments. (right) Relative expression levels of wild-type and pseudoexon-containing transcripts by qRT-PCR. The ratio between the two isoforms in samples silenced for either hnRNP F or H was also calculated. (B) Transient overexpression of hnRNP F. (left) GeneMapper windows displaying fluorescence peaks corresponding to RT-PCR products obtained from the cDNA of cells transfected with constructs expressing the M minigene with or without hnRNP F overexpression. The fluorescence peak areas were measured by the GeneMapper v4.0 software. The X-axis represents data points (size standard peaks are also indicated) and the Y-axis represents fluorescence units. (right) Histograms represent the relative amount of transcripts including or skipping the pseudoexon, as assessed by calculating the ratio of the corresponding fluorescence peak areas (setting the sum of all peaks as 100%). Bars represent mean ± SD of 3 independent experiments, each performed in triplicate. The results were analyzed by unpaired t-test (*P<0.05; **P<0.01; ***P<0.001).

As a first step in the study of regulatory elements controlling pseudoexon inclusion, we analyzed the 75-bp pseudoexon sequence and noticed the presence of three G-runs motifs (named G1, G2, and G3): two are located at the 5′ of the pseudoexon (positions −1 to +4 and +13/15), the third towards the 3′ end (position 60–62) (Figure 1). As hnRNP H and F are known to bind G-runs, acting either as splicing-enhancer or splicing-inhibitory factors depending on gene and cellular context [8], [18], [19], we explored their effect on *FGG* pseudoexon inclusion by performing siRNA-mediated silencing of the two proteins (Figure 2A). The pT-*FGG*-IVS6-320A>T minigene (containing the mutant IVS6-320A>T *FGG* genomic region spanning 1,858 bp from intron 4 to intron 7, cloned into the pTargeT vector) [17] was thus co-transfected into HeLa cells (not expressing fibrinogen) with siRNAs against hnRNP F or hnRNP H. The efficacy of protein knockdowns was verified and quantitated by Western blotting (Figure 2A, left and central panels). Interestingly, real-time RT-PCRs showed that knockdown of hnRNP H results in a non-significant increase of pseudoexon inclusion, whereas hnRNP F depletion significantly represses pseudoexon recognition (Figure 2A, right panel). A similar result was found after double knockdown of hnRNP F and H (data not shown), suggesting a prominent role of hnRNP F in the modulation of *FGG* pseudoexon splicing. The lack of response to hnRNP H might raise the question whether a sufficient level of knockdown of this protein was obtained. However, silencing of hnRNP H was performed using exactly the same protocol and reaching the same level of silencing (85%) that we previously showed to determine the activation of a cryptic acceptor splice site in the thrombopoietin gene [20].

In complementary experiments, the overexpression of hnRNP F resulted in a further decrease of the wild-type residual transcript, from about 5% to about 1% of the total FGG transcripts, thus confirming the role of this factor in promoting pseudoexon inclusion (Figure 2B). In this case, to have a more accurate measure of the relative amount of the two splicing variants, a fluorescent RT-PCR approach was used (see Materials and Methods).

### A 25-bp Region Binds hnRNP F and is Important for Pseudoexon Inclusion

**Figure 3:**
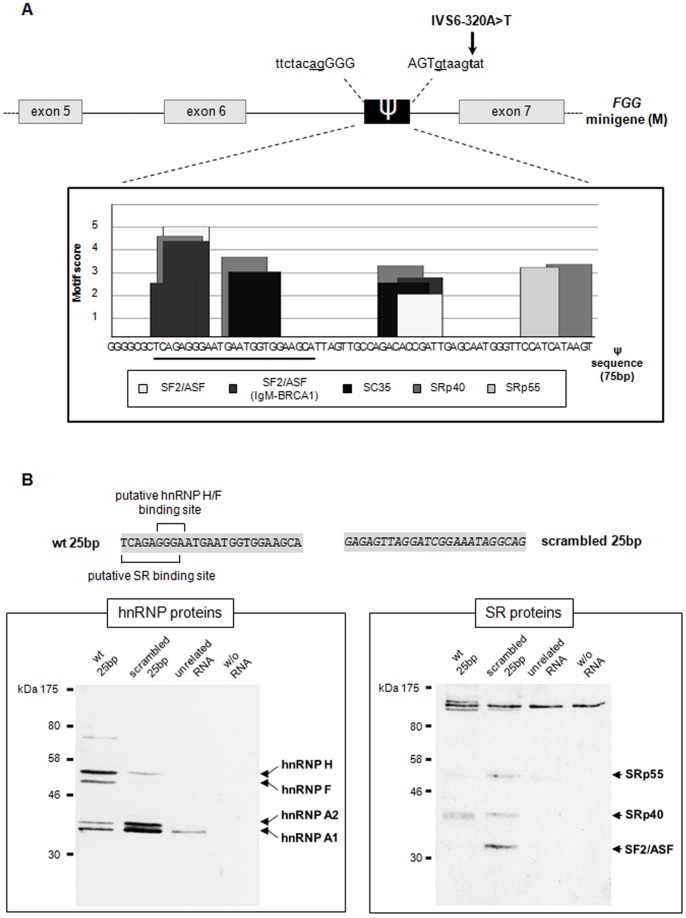
*In-silico* prediction of an ESE-enriched 25-bp sequence and identification of the interacting proteins. (A) Schematic representation of the minigene construct containing the 75-bp *FGG* pseudoexon activated by the IVS6-320A>T mutation (indicated by an arrow). (bottom) ESE elements predicted by the ESEFinder program (http://rulai.cshl.edu/cgi-bin/tools/ESE3/esefinder.cgi?process=home) in the pseudoexon sequence are indicated by the histogram bars; the ESE-enriched 25-bp sub-region is underlined. (B) Western blot analysis of RNA-protein pulldown assays for the identification of *trans*-acting factors binding to the 25-bp region. (top) Sequences used in pulldown experiments. (bottom) Western blot analysis of hnRNP and SR proteins immunoprecipitated from HeLa nuclear extracts with either the wild-type or the scrambled 25-bp (as negative control) pseudoexon sequence.

Besides G-runs, additional regulatory elements modulating pseudoexon inclusion were predicted using the ESEfinder software [21], . The program predicted multiple binding sites for SF2/ASF, SC35, SRp40, and SRp55 proteins. The higher density of high-score motifs (score >3) was found in a 25-bp region comprised between nucleotides 7 and 27 of the pseudoexon (Figure 3A), this region also contains one of the three G-run motifs that may bind hnRNP F. To identify which *trans*-acting factors can bind this ESE-enriched 25-bp region, an affinity pulldown protocol was used. Both the wild-type and a scrambled (negative control) 25-bp sequence (Figure 3B), were covalently coupled to adipic acid dehydrazide beads and incubated with HeLa nuclear extracts. As shown in Figure 3B (left panel), Western blotting with antibodies against the principal hnRNPs evidenced signals for all four tested proteins (hnRNP H, F, A1, and A2). However, comparison with results obtained using a scrambled or an unrelated oligoribonucleotide revealed that the only protein exclusively binding the 25-bp target sequence was hnRNP F. Moreover, the binding efficiency of hnRNP H to the 25-bp probe was much higher than that of the permutated sequence. The same experiment performed immunodecorating with antibodies against SR proteins evidenced a weak binding of SRp40 to the 25-bp sequence (Figure 3B, right panel), supporting the ESEfinder prediction for this protein. Finally, probing with additional antibodies (i.e. anti PTB, hnRNP C) ruled out the binding of additional common hnRNP factors to this sequence (data not shown).

**Figure 4:**
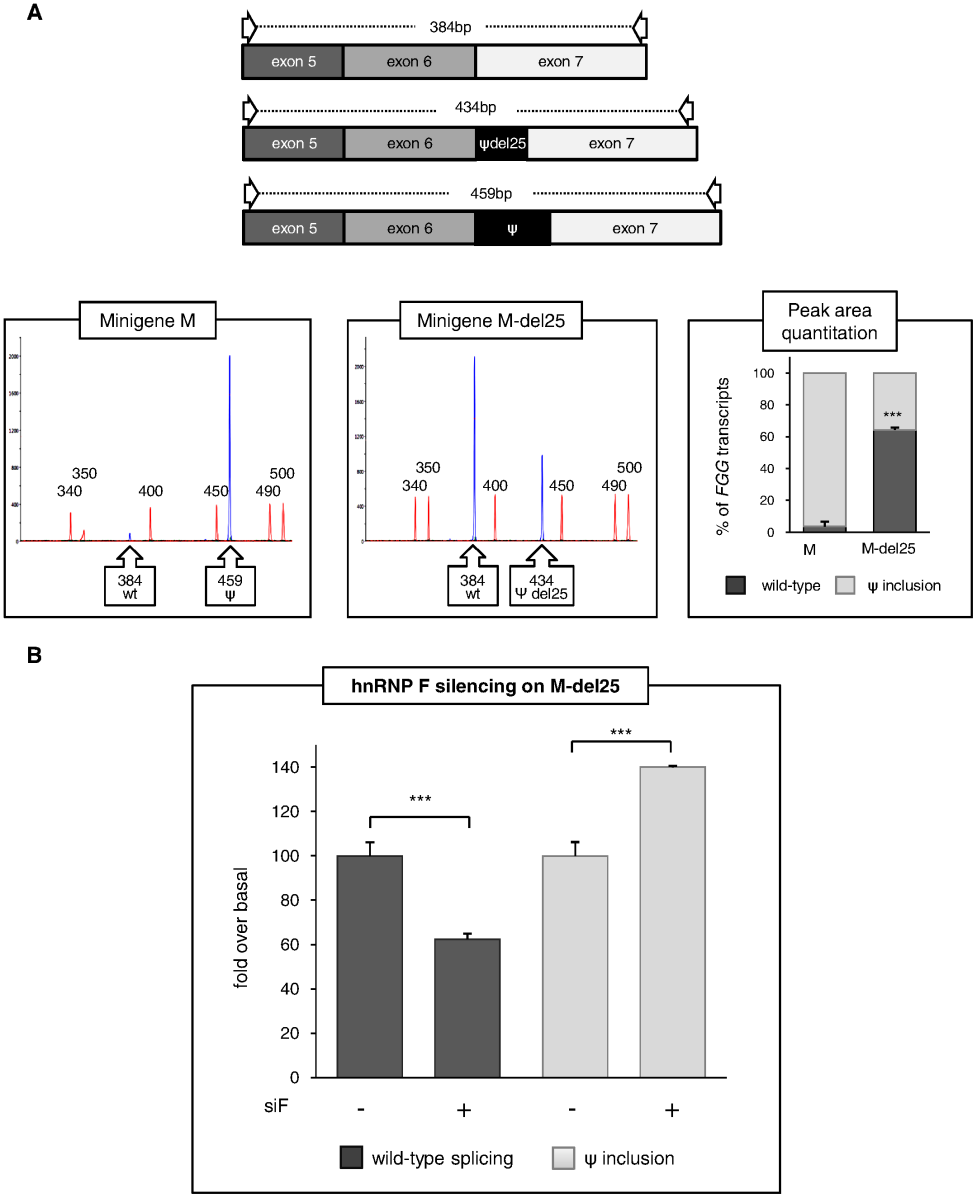
Functional characterization of the 25-bp region. (A) Effect of the 25-bp region on pseudoexon inclusion. Minigene constructs either containing (M) or lacking (M-del25) the 25-bp region were transiently transfected in HeLa cells. The relative amount of pseudoexon inclusion was measured by fluorescent RT-PCR. (top) Schematic representation of the RT-PCR products; primers used in RT-PCR experiments are indicated by arrows. The length of each fragment is also indicated. (bottom, left and middle panels) GeneMapper windows displaying fluorescence peaks corresponding to the RT-PCR products. The fluorescence peak areas were measured as described in Figure 2B legend. (bottom, right panel) Histograms representing the relative amount of transcripts including or skipping the pseudoexon, as assessed by calculating the ratio of the corresponding fluorescence peak areas (setting the sum of all peaks as 100%). Bars represent mean ± SD of 3 independent experiments, each performed in triplicate. (B) Knock-down experiments showing that silencing of hnRNP F in the absence of the 25-bp region significantly promotes pseudoexon inclusion. Quantitation by qRT-PCR demonstrates that the hnRNP F splicing-enhancer activity is dependent on the integrity of the 25-bp region. The results were analyzed by unpaired t-test (***P<0.001).

The relevance of the 25-bp region in promoting pseudoexon inclusion was experimentally verified by deleting this sequence in the pT-*FGG*-IVS6-320A>T plasmid. Transient transfection of the 25-bp-deleted construct (pT-*FGG*-M-del25) in HeLa cells resulted in a change in pseudoexon inclusion from 96% to 44%, as quantified by fluorescent RT-PCR (Figure 4A). The marked reduction in pseudoexon inclusion confirmed that the deleted nucleotides are necessary to reach full efficiency in pseudoexon recognition. Similar results were obtained by qRT-PCR analysis (see Figure S2).

To confirm that hnRNP F acts by interacting with the 25-bp region, hnRNP F silencing was performed in cells expressing the pT-FGG-M-del25 plasmid. In contrast with what observed in the presence of the whole pseudoexon sequence (see Figure 2A), silencing of hnRNP F in the absence of the 25-bp region significantly promoted pseudoexon inclusion (Figure 4B). This result suggests that: 1) the role of hnRNP F in enhancing pseudoexon recognition is strictly dependent on the presence of the 25-bp region; 2) the two G-run motifs located outside this region may act as ESSs.

Since the predicted hnRNP F binding site within the 25-bp region is partially overlapped to a SRp40 binding site (Figure 3A) and that indeed a weak binding of SRp40 was evidenced by pulldown experiments (Figure 3B), we attempted to modulate SRp40 level in HeLa cells. While we could not reach a sufficient level of SRp40 silencing, overexpression experiments showed that, at least in our experimental conditions/system, the percentage of pseudoexon inclusion is insensitive to SRp40 upregulation (Figure S3).

### Different G-run Elements Exhibit Opposite Effects in Regulating Pseudoexon Inclusion

**Figure 5:**
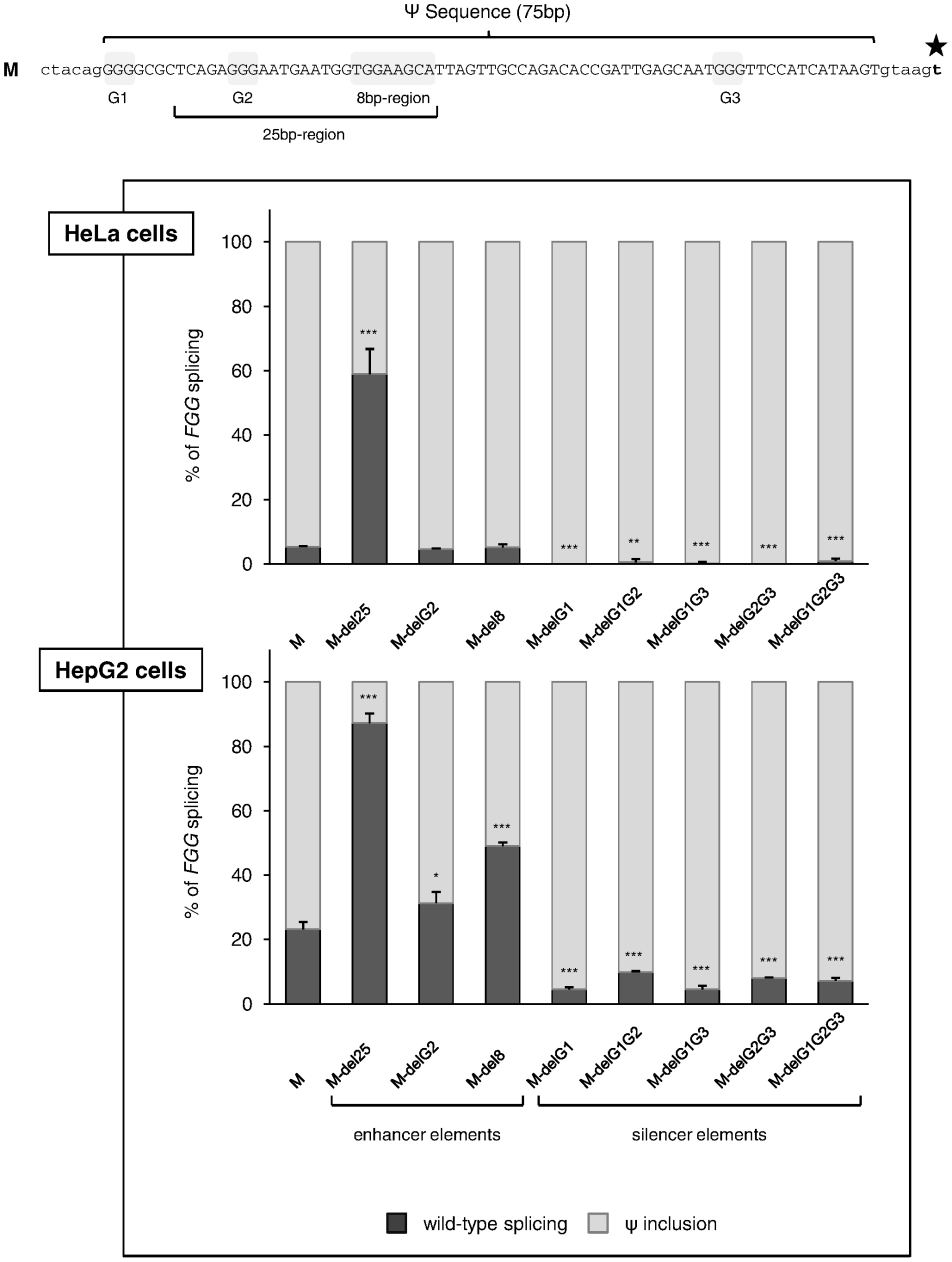
Functional dissection of G-run elements within the pseudoexon sequence. (top) The complete 75-bp-long pseudoexon sequence and flanking splice sites; nucleotides belonging to the pseudoexon are in capital letters; the star indicates the IVS6-320A>T mutation; the deleted sequences (shaded in gray) are indicated. (bottom) Histograms representing the relative amount of transcripts including or skipping the pseudoexon, calculated for each deletion mutant as described in the legend of Figure 2B. Bars represent mean ± SD of 3 independent experiments, each performed in triplicate. The results were analyzed by unpaired t-test. Statistical significance was calculated referring to the M construct (*P<0.05; **P<0.01; ***P<0.001).

To further dissect the functional elements within the splicing-promoting 25-bp region, as well as to map all hnRNP F binding sites within the pseudoexon sequence at a higher resolution, we decided to test the effect of the single (G1 and G2) and combined (G1+G2, G1+G3, G2+G3, and G1+G2+G3) deletion of the three different G-runs in the pT-*FGG*-IVS6-320A>T plasmid. Moreover, as pseudoexon regulation might depend on the cellular context, transient transfections of the mutant constructs were performed also in human hepatoma HepG2 cells, which endogenously express fibrinogen and therefore represent a more physiological model system than HeLa. Experiments in HepG2 showed no physiological expression of transcripts including the *FGG* pseudoexon (data not shown), and a higher level of *FGG* wild-type splicing (23%) in the presence of the IVS6-320A>T mutation, thus allowing a more accurate analysis of the effects of the different deletion constructs (Figure 5).

In HepG2, the expression of the G2-deleted construct (pT-*FGG*-M-delG2), lacking the only G-run element located within the 25-bp region, resulted in a significant reduction in pseudoexon inclusion (from 77% to 68%) (Figure 5), confirming our hypothesis that this hnRNP binding site functions as an ESE. Surprisingly, the ablation of G2 had no effect on splicing in HeLa cells, indicating a certain degree of cell type-specific responsiveness of this element. This discrepancy might be due either to differences in the basal level of expression of hnRNP F between the two analyzed cell lines, or to an additional trans-acting factor only present in HepG2. The first possibility was explored by real-time RT-PCR and Western blot assays, showing significantly lower levels of both endogenous hnRNP F mRNA and protein in HepG2 compared to HeLa cells (Figure S4).

Contrary to what was observed for delG2 mutants, all single or combined deletions of G-runs outside the 25-bp region resulted in a marked increase of pseudoexon splicing in both cell types, suggesting that G1 and G3 normally act as repressor elements (Figure 5).

As the deletion of the G2 element alone does not completely recapitulate the effect of the ablation of the entire 25-bp region, and considering that hnRNP F has three RNA-recognizing motifs arrayed in the same spacing that can bind to extended purine-rich elements [23], we produced an additional deleted construct (pT-*FGG*-M-del8) lacking the very last 8-bp purine-rich sequence of the 25-bp region. This mutant was transfected in both HeLa and HepG2 cells again showing a cell-type specific response (Figure 5). In particular, in HepG2 cells the ablation of the 8-bp element led to a significantly lower inclusion of the pseudoexon (from 77% to 51% of total *FGG* transcripts).

Taken together, these results demonstrate that G-runs with opposite functions contribute in determining the levels of pseudoexon inclusion in *FGG* transcript, only in the presence of the IVS6-320A>T mutation.

## Discussion

Detailed knowledge on the structure of most vertebrate genes has highlighted the presence of a large number of pseudoexon sequences that are physiologically silenced by intrinsically defective splice sites [24], by the presence of silencer elements [25], [26], [27], or by the formation of inhibiting RNA secondary structures [28]. Even though pseudoexons are expected to be low in ESEs, the high degeneration of splicing enhancer motifs and their relative abundance also within introns [5] suggest that pseudoexons probably contain also a number of enhancer motifs, which can exert their splicing-promoting activity when one of the above-mentioned repressing conditions is abolished. However, the complex interactions among regulatory mechanisms controlling pseudoexon splicing are still largely unexplored.

In this work, we made use of a mutation-activated pseudoexon in the *FGG* gene, previously identified by our group, to investigate the *cis*- and *trans*-acting factors controlling pseudoexon inclusion in the mature transcript.

A combination of bioinformatics predictions and functional analyses of regulatory sequences led to the identification of a 25-bp region, whose presence is important for pseudoexon inclusion. This region contains both an hnRNP H/F (G-run motif) and several putative SR-protein binding sites, which were functionally characterized by performing pulldown experiments and modulating the levels of the corresponding *trans*-acting interactors.

The strength and specificity of the binding of SR and hnRNP proteins to the 25-bp region, suggested that hnRNP H/F might represent important regulatory factors, possibly through the interaction with a G-run element at position +13/15 of the pseudoexon sequence. Even though these *trans*-acting splicing regulators are generally regarded as splicing repressors, recent works suggested that their role is probably much more complex. Indeed, a large survey on the contribution of hnRNP A1 and H on alternative splicing revealed that these proteins can stimulate both exon inclusion and skipping events [29]. To test whether hnRNP H and F modulate pseudoexon inclusion in the mature transcript, both proteins were knocked down by siRNAs in HeLa cells. Silencing of hnRNP F unexpectedly resulted in decreased pseudoexon inclusion, indicating that, in this position, it can act as a positive modulator of *FGG* pseudoexon splicing.

In addition, unlike other genes in which hnRNP H and F have overlapping functions *in vivo* [18], [19], [30], our results suggest that these two proteins are not completely redundant in regulating *FGG* pseudoexon inclusion. Indeed, while knockdown of hnRNP F decreases pseudoexon inclusion, silencing of hnRNP H does not significantly alter *FGG* splicing, although both proteins are silenced at similar levels (Figure 2). Partially overlapping but non-redundant function of hnRNP H and F has also been reported for the DM20 gene [31].

Considering that the 75-bp pseudoexon includes two additional G-runs, i.e. a gGGGG stretch at position -1/+4 (G1) and a GGG motif at position +60/63 (G3) (Figure 1), the specific contribution of hnRNP F binding to the G-run motif within the 25-bp region (G2) was analyzed by RNAi experiments in cells transfected with the 25-bp-deleted minigene. In the absence of this region, hnRNP F silencing led to a significant increase in pseudoexon inclusion, confirming that the splicing-enhancing activity of hnRNP F in the wild-type pseudoexon sequence is specifically mediated by the 25-bp region.

Although promising, the results obtained using the 25-bp-deleted minigene -in which the pseudoexon has been shortened to 50 bp- might be partially affected by the intrinsic tendency of skipping of small exons [32]. To avoid this confounding effect and to confirm the splicing-enhancing activity of the 25-bp region, a fine mapping of specific enhancer elements was performed. In particular, further dissection of hnRNP F binding sites within the whole *FGG* pseudoexon, by multiple small deletions removing the G-run elements, nicely confirmed that the G2 element is a splicing enhancer, whereas the G1 and G3 motifs act as canonical ESS (Figure 5). Moreover, a second functional purine-rich element within the 25-bp region was found, which might cooperate with the enhancer G2-run in hnRNP F responsiveness. Interestingly, the two identified ESEs were functional only in HepG2 cells, revealing a cell-type specific regulation of pseudoexon splicing. This might be dependent, at least in part, on the difference in hnRNP F levels between the two analyzed cell models (Figure S4A), although the involvement of additional hepato-specific *trans*-acting factors cannot be ruled out. Indeed, when hnRNP F was overexpressed in HepG2, we obtained, for all M mutant constructs, a response more similar to that obtained in HeLa in basal condition (Figure S4B), suggesting that the fine regulation of splicing factor levels in different cell lines is important to modulate the amount of pseudoexon recognition by the splicing machinery. Concerning the question of how the G-runs or hnRNP F increase exon-definition, recent reports have suggested that G-stretches near to donor sites may act co-operatively to recruit U1snRNA, either through direct binding or through other splicing factors [33]. This effect, however, was shown to be critically dependent on the proximity of the G-run to the splice site. In our case, considering the distance between the G2 element and the 5′ splice site of *FGG* pseudoexon, this possibility is rather unlikely. Alternatively, we can speculate that our G-runs may also mediate direct binding of U2AF35 to the 3′ splice site of the pseudoexon, a possibility that would be interesting to test in the future.

Taken together, these results highlight several important issues with regards to splicing regulation. First of all, the importance of always checking experimentally, whenever possible, the *trans*-acting factors binding to *in-silico* predicted elements. Although *in-silico* methods are constantly improving, there is still a major gap between predicted and actual binding sites, as shown in our pulldown experiments. A second consideration regards the importance of not making too close a parallelism between the presence of a specific regulator and its potential effects on the inclusion (or exclusion) of any pre-mRNA sequence in the mature transcript. Indeed, our data suggest that hnRNP F can act either as activator or as repressor of pseudoexon inclusion through the binding of different *cis*-acting elements.

Finally, this study highlights the intrinsic complexity of the splicing process, even in sequences that are not subjected to evolutionary pressure (Figure S1B).

## Materials and Methods

### Plasmids

The minigene construct pT-*FGG*-IVS6-320A>T, containing the mutant IVS6-320A>T human *FGG* genomic region spanning from intron 4 to intron 7 (based on GenBank accession number NG_008834), was previously described [17]. The deletion mutants (M-del25, M-del8, M-delG1, M-delG2, M-delG1G2, M-delG1G3, M-delG2G3, M-delG1G2G3) were produced by site-directed mutagenesis with oligonucleotides (Sigma-Aldrich, St Louis, MO, USA; sequences available upon request) carrying the nucleotide deletion. For the M-del25 mutant, the mutagenesis reaction was carried by using a slight modification of the QuickChange Site-Direct Mutagenesis Kit protocol (Agilent Technologies Inc, Santa Clara, CA, USA), consisting in the use of longer primers (50 nucleotides) bridging the deletion site. All constructs were purified by the EndoFree Plasmid Maxi Kit (Sigma-Aldrich) and checked by DNA sequencing using the BigDye Terminator Cycle Sequencing Kit v1.1 and an automated ABI-3130XL DNA sequencer (Life Technologies, Carlsbad, CA, USA).

The plasmid pCG-SRp40 used for overexpression experiments was previously described [34]. The pCDNA3-hnRNP F plasmid for hnRNP F overexpression was obtained by cloning the amplified cDNA sequence of this protein (AAH01432) in the commercial pcDNA3 vector (Life Technologies).

### Cell Culture, Transfection, and RNA Extraction

Human cervix carcinoma HeLa cells were maintained in Dulbecco modified Eagle’s medium (EuroClone, Milan, Italy), HepG2 cells were cultured in RPMI 1640 (EuroClone) additioned with sodium pyruvate (1 mM; Sigma-Aldrich). Both media were supplemented with 10% fetal bovine serum, 1% glutamine, and antibiotics (100 U/mL penicillin and 100 µg/mL streptomycin; EuroClone). Cells were grown at 37°C in a humidified atmosphere of 5% CO_2_ and 95% air, according to standard procedures.

In each transfection experiment, an equal number of cells (250,000) were transiently transfected in 6-well plates with the Fugene HD reagent (Promega, Madison, WI, USA) and 4 µg of plasmid DNA, following the manufacturer’s instructions. Twenty-four hours after transfection, cells were washed twice with phosphate-buffered saline and total RNA was extracted by using the EUROzol reagent (EuroClone), according to the manufacturer’s instructions.

### RNA Interference

For hnRNP H and F knockdown 150,000 HeLa cells were seeded on 3.5-cm multiwell plates. After 24 hours, 5 µL Oligofectamine (Life Technologies) were mixed with 15 µL Opti-MEM I reduced serum medium (Life Technologies), incubated at room temperature for 7 minutes and added to 2.5 µL (25 pmol) of siRNA duplex (10 µM), which had been mixed with 175 µL Opti-MEM I. The mixture was incubated at room temperature for 20 minutes, and then added to the cells. After 24 hours, effector and reporter constructs were transfected as described above. Cells were grown for an additional 24 hours followed by RNA and protein extraction. The 20-nt target sequences in hnRNP H and F were 5′-GGAAATAGCTGAAAAGGCT-3′ and 5′-GCGACCGAGAACGACATTT-3′, respectively. A pre-designed siRNA targeting luciferase (Target Sequence: 5′-CGTACGCGGAATACTTCGA-3′) (EuroClone) was used as negative control. Silencing efficiency was assessed by Western blotting performed according to standard protocols. The effect of siRNA treatment against hnRNP H and F on pseudoexon inclusion was assessed by real-time RT-PCR with transcript-specific amplicons, as further detailed.

### Real-time RT-PCR

Random nonamers and ImProm-II Reverse Transcriptase System (Promega) were used to perform first-strand complementary DNA (cDNA) synthesis starting from 1 µg of total RNA, according to the manufacturer’s instructions. Two primer couples were designed in order to be specific for transcripts containing or lacking the pseudoexon; qRT-PCR reactions (20 µL) were performed using the 2x SYBR green master mix (Roche, Basel, Switzerland) in a Light Cycler 480 (Roche). Oligonucleotide sequences and cycling conditions are available on request. The percentage of pseudoexon inclusion was calculated as the ratio between the relative quantitation of the amplicon including the pseudoexon (normalized by the ΔCt method, using as reference gene an intron-containing transcript produced by the pTargeT vector itself) and the relative quantitation of the skipped transcript (normalized as described for the pseudoexon-containing transcript). Melting-curve analysis was used to verify that a single product had been amplified in each real-time reaction.

### Fluorescent RT-PCR

To quantify splice products, an aliquot (1 µL) of the total reverse-transcription reaction (20 µL) was used as template in a standard RT-PCR amplification using a fluorescein-labeled exonic forward primer (*FGG*–Ex5-F-FAM: 5′-[6FAM]AGAAGGTAGCCCAGCTTGA-3′) and the exonic reverse oligonucleotide *FGG*–Ex7-R (5′-ATTCCAGTCTTCCAGTTCCA-3′). For experiments in HepG2, the reverse primer was substituted with the commercial pTargeT sequencing primer (Promega) to discriminate transcripts produced by the transfected construct from the endogenous *FGG* mRNA. PCRs were carried out under standard conditions using the FastStart Taq DNA Polymerase (Roche) on a Mastercycler EPgradient (Eppendorf AG, Hamburg, Germany). PCR reactions were separated on an ABI-3130XL sequencer and the peak areas measured by the GeneMapper v4.0 software. The level of pseudoexon inclusion was assessed by measuring the ratio of the fluorescence peak areas corresponding to the transcript including or skipping the pseudoexon. Because the two PCR products are amplified by the same primers, and the two amplicons have similar amplification efficiencies (as assessed by generating standard curves for each amplicon using real-time PCR, data not shown), the ratio of amplified products reflects the relative abundance of the templates before PCR.

### Pulldown Protocol

RNA probes containing the wild-type 25-bp region and a scrambled version of the same sequence (as negative control) were obtained by chemical synthesis from Sigma-Aldrich; as control of the RNA precipitation an unrelated small RNA oligonucleotide was used (wild type ATM 5′-UGGCCAGGUAAGUGAUAUAU-3′) [35]. The pulldown protocol has been described in detail by Sevo and colleagues [36]. Briefly, 500 pmoles of the target RNA were placed in a 400-µL reaction mixture containing 100 mM NaOAC pH 5.0 and 5 mM sodium m-periodate (Sigma-Aldrich), incubated for 1 hour in the dark at room temperature, ethanol precipitated, and resuspended in 100 µL of 0.1 M NaOAC, pH 5.0. To this RNA, 100 µL of adipic acid dehydrazide agarose bead (50% slurry, Sigma-Aldrich) equilibrated in 100 mM NaOAC pH 5.0 were added, and the mix was incubated for 12 hours at 4°C on a rotator. RNA beads were then washed with 2 M NaCl and equilibrated in washing buffer (5 mM HEPES pH 7.9, 1 mM MgCl_2_, 0.8 mM magnesium acetate). The beads were then incubated on a rotator with a protein mixture containing approximately 1 mg of HeLa cell nuclear extract (Cil Biotech, Mons, Belgium) for 30 minutes at room temperature in 1 mL final volume. The beads were subsequently pelleted by centrifugation at 3000 rpm for 3 minutes and washed 4 times with 1.5 mL of washing buffer, before addition of sodium dodecyl sulfate (SDS) sample buffer and loading on a 10% SDS-PAGE gel. The samples were analyzed by Western blotting with a general antibody against SR proteins (1H4, Zymed Laboratories, San Francisco, CA, USA) and several home-made antibodies against hnRNP A/B and hnRNP H/F, previously described in our studies [37].

## Supporting Information

Figure S1 **Analysis of** ***FGG*** **pseudoexon donor splice site and overall sequence conservation.** (A) Comparison of cryptic donor splice site of the pseudoexon with all the sequences of the physiologic donor sites in *FGG* exons. (B) UCSC snapshot showing the alignment of the 75-bp *FGG* pseudoexon sequence in vertebrates.
(TIF)

Figure S2 **Effect of the 25-bp-region removal on pseudoexon inclusion by qRT-PCR.** (left) Minigene constructs either containing (M) or lacking (M-del25) the 25-bp region transiently transfected in HeLa cells. (right) Relative expression levels of wild-type and pseudoexon-containing transcripts, and ratio between the two isoforms in cells expressing M and M-del25 plasmid, evaluated by qRT-PCR. Bars represent mean ± SD of 3 independent experiments, each performed in triplicate. The results were analyzed by unpaired t-test (**P<0.01; ***P<0.001).
(TIF)

Figure S3 **Effect of SRp40 overexpression on the** ***FGG*** **pseudoexon splicing in HeLa cells.** RT-PCRs were performed on cDNA of cells co-transfected with the SRp40 and the minigene M constructs by using a FAM-labeled primer. RT-PCR products were separated by capillary electrophoresis on a 3130XL genetic analyzer. Histograms represent the relative amount of transcripts including or skipping the pseudoexon, as assessed by calculating the ratio of the corresponding fluorescence peak areas (setting the sum of all peaks as 100%). Bars represent mean ± SD of 3 independent experiments, each performed in triplicate. The results were analyzed by unpaired t-test.
(TIF)

Figure S4 **Differences in hnRNP F expression might account for cell-specific inclusion levels of** ***FGG*** **pseudoexon.** (A) Western blot (left) and corresponding densitometric analysis (middle) demonstrating the endogenous expression levels of hnRNP F protein in both HeLa and HepG2 cells. (right) Relative expression levels of hnRNP F mRNA by qRT-PCR in the two cell lines. (B) Histograms representing the relative amount of transcripts including or skipping the pseudoexon, calculated by fluorescent RT-PCR for each deletion mutant after overexpression of hnRNP F in HepG2 cells. Bars represent mean ± SD of 3 independent experiments, each performed in triplicate. The results were analyzed by unpaired t-test. Statistical significance was calculated referring to the M construct (***P<0.001).
(TIF)
