# Supplementary figures and images for: Dual Role of G-runs and hnRNP F in the Regulation of a Mutation-Activated Pseudoexon in the Fibrinogen Gamma-Chain Transcript
Source: PLoS One. 2013 Mar 22;8(3):e59333. doi: 10.1371/journal.pone.0059333 (PMC3606458; doi:10.1371/journal.pone.0059333)

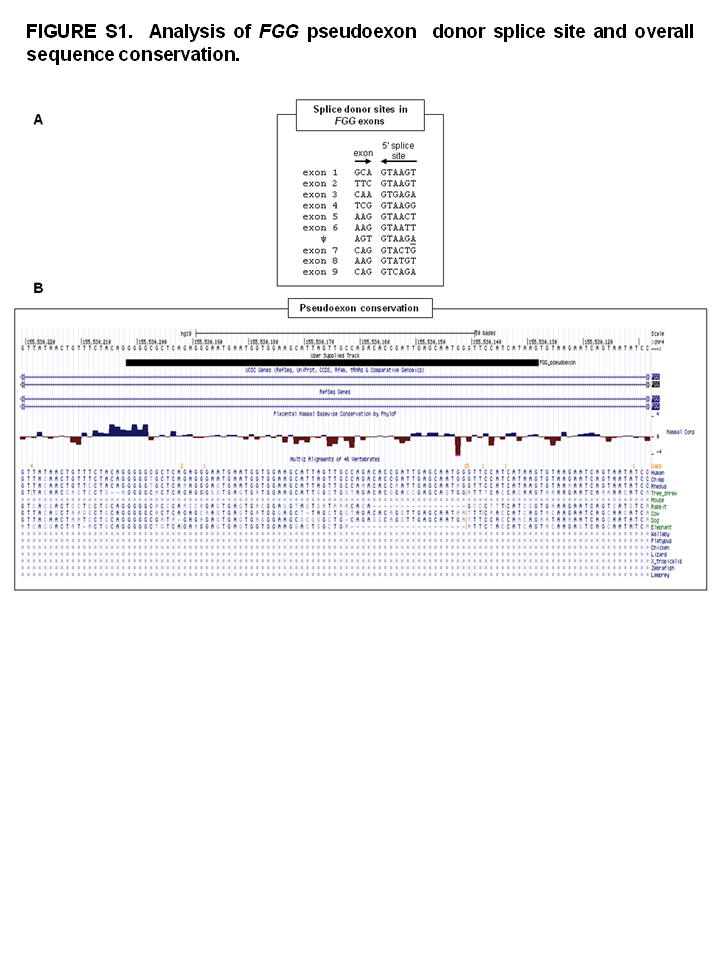

Supplement: Figure S1 — Analysis of FGG pseudoexon donor splice site and overall sequence conservation. (A) Comparison of cryptic donor splice site of the pseudoexon with all the sequences of the physiologic donor sites in FGG exons. (B) UCSC snapshot showing the alignment of the 75-bp FGG pseudoexon sequence in vertebrates. (TIF) [file pone.0059333.s001.tif]
